# Xenon attenuated neonatal lipopolysaccharide exposure induced neuronal necroptosis and subsequently improved cognition in juvenile rats

**DOI:** 10.3389/fphar.2022.1002920

**Published:** 2022-12-02

**Authors:** Zhimin Liao, Xiaofeng Ou, Cheng Zhou, Daqing Ma, Hailin Zhao, Han Huang

**Affiliations:** ^1^ Department of Anesthesiology, Key Laboratory of Birth Defects and Related Diseases of Women and Children, West China Second University Hospital, Sichuan University, Chengdu, China; ^2^ Department of Anesthesiology and Translational Neuroscience Center, West China Hospital, Sichuan University, Chengdu, China; ^3^ Anaesthetics, Pain Medicine, and Intensive Care, Department of Surgery and Cancer, Faculty of Medicine, Imperial College London, Chelsea and Westminster Hospital, London, United Kingdom

**Keywords:** neonatal sepsis, xenon, neurodevelopmental impairment, necroptosis, neuroinfl ammation

## Abstract

**Background:** Neonatal sepsis is known to cause neurodevelopment impairment and has been reported to increase risks for neurological/psychiatric disorders. In this study, we investigated the effect of xenon, a well-known potent neuroprotective gas, on neonatal sepsis-induced neurodevelopment impairment in rats together with underlying mechanism by focusing on receptor-interacting protein kinase (RIP) mediated neuronal necroptosis.

**Methods:** 3-day-old Sprague–Dawley rat pups were exposed to either 70% xenon or N_2_ balanced with O_2_ for 6 h, during which lipopolysaccharide (LPS) was injected intraperitoneally for 3 times (500 μg/kg for the 1st and 250 μg/kg for the second and third dose; *n* = 6–10/group). In another cohort of 3-day-old rat pups, intracerebroventricular injection of necrostatin-1 (4 µg in 4 µl saline, a RIP-1-targeted inhibitor of necroptosis) was performed 20 min after the third dose of LPS. The learning ability and memory were assessed 25 days after LPS injection. Then, their hippocampus was collected for neuronal necroptosis with RIP and MIKL assessments using western blot and *in situ* immunostaining. Systemic and neuro-inflammation was also assessed.

**Results:** LPS insult resulted in elevation of pro-inflammatory cytokine TNF-𝝰 and IL-6, caused neuronal necroptosis and damaged synaptic integrity at the brain developing stage, which finally led to the long-term cognitive impairment. Xenon inhibited necroptosis associated mediator RIP-1, RIP-3, and MLKL activation, protected neurons and attenuated cognitive dysfunction induced by LPS. Like xenon, the similar pattern changes induced by a RIP-1 inhibitor Necrostatin-1 were also found.

**Conclusion:** This study indicates that necroptosis is involved in neonatal sepsis-induced neurofunctional impairments and xenon may be a novel therapeutic strategy to prevent/treat cognitive impairment in neonatal septic patients.

## Introduction

The newborns are at high risks for serious infections followed by sepsis, which is one of the leading causes of newborn deaths and disability globally (Black et al., 2010; Chau et al., 2012; Liu et al., 2012). Furthermore, neonatal sepsis has been identified as one of the risk factors for development of neurological and psychiatric disorders (Stoll et al., 2004; Schlapbach et al., 2011; Mitha et al., 2013) in juvenile or early adulthood but the underlying mechanisms remain largely unknown. Although sepsis and subsequent neuroinflammation are likely to be initial key triggers, the long-term outcome of the neurodevelopmental impairment is complicated. This is because: 1) the onset of neurodevelopment impairments takes years or even decades; and 2) antibiotic therapy is not sufficient to prevent brain injury associated with neonatal sepsis. Therefore, it is rationalized that other factors derived from initial inflammatory response may be involved in the development of neonatal sepsis-induced neurological/psychiatric disorders.

Necroptosis, a programmed form of necrosis (Pasparakis and Vandenabeele, 2015), has been implicated in the pathogenesis of organ injuries, such as ischemia-reperfusion injury (Lau et al., 2013; Linkermann and Green, 2014; Pavlosky et al., 2014). Tumor necrosis factor alpha (TNF-α)-induced necroptosis has been extensively investigated. Upon TNF-α stimulation, the apoptotic machinery, FADD, c-FLIP and caspase-8, suppresses the induction of necroptosis (Vanlangenakker et al., 2011). However, once caspase-8 is inactivated due to various reasons, receptor interacting protein-1 (RIP-1) then interacts with RIP-3, which promotes the phosphorylation of mixed lineage kinase domain-like protein (MLKL) (Li et al., 2012; Cai et al., 2014). Phosphorylated MLKL forms tetramers and translocates into the plasma membrane (Sun et al., 2012) and, in turn, initiates Ca2+ influx and the cell undergoes necroptosis (Cho et al., 2009). Inhibition of key molecules in necroptosis, such as RIP-1 or RIP-3, confers potent protection against various solid organ injuries in which inflammatory response is heavily involved (Northington et al., 2011; Re et al., 2014; Zhang et al., 2016). Interestingly, the noble gas xenon has also been shown to be protective in several models of inflammation-related organ injuries, such as anesthetic-induced cell death in the developing brain (Shu et al., 2010) and lung injury after renal transplant (Zhao et al., 2015).

The present study was undertaken to investigate whether inhalation of xenon gas ameliorates neonatal sepsis-induced neurodevelopmental impairment at early adulthood age in rats via modulation of apoptotic and/or necroptotic cell death pathways.

## Material and methods

This animal study was approved by the Institutional Animal Experimental Ethics Committee of Sichuan University (Chengdu, Sichuan, China). All the experiments were carried out in accordance with the National Institutes of Health guide for the care and use of Laboratory animals (NIH Publications No. 8023, revised 1978) and the study protocol complies with the ARRIVE guidelines.

### Lipopolysaccharide administration and xenon exposure

**FIGURE 1:**
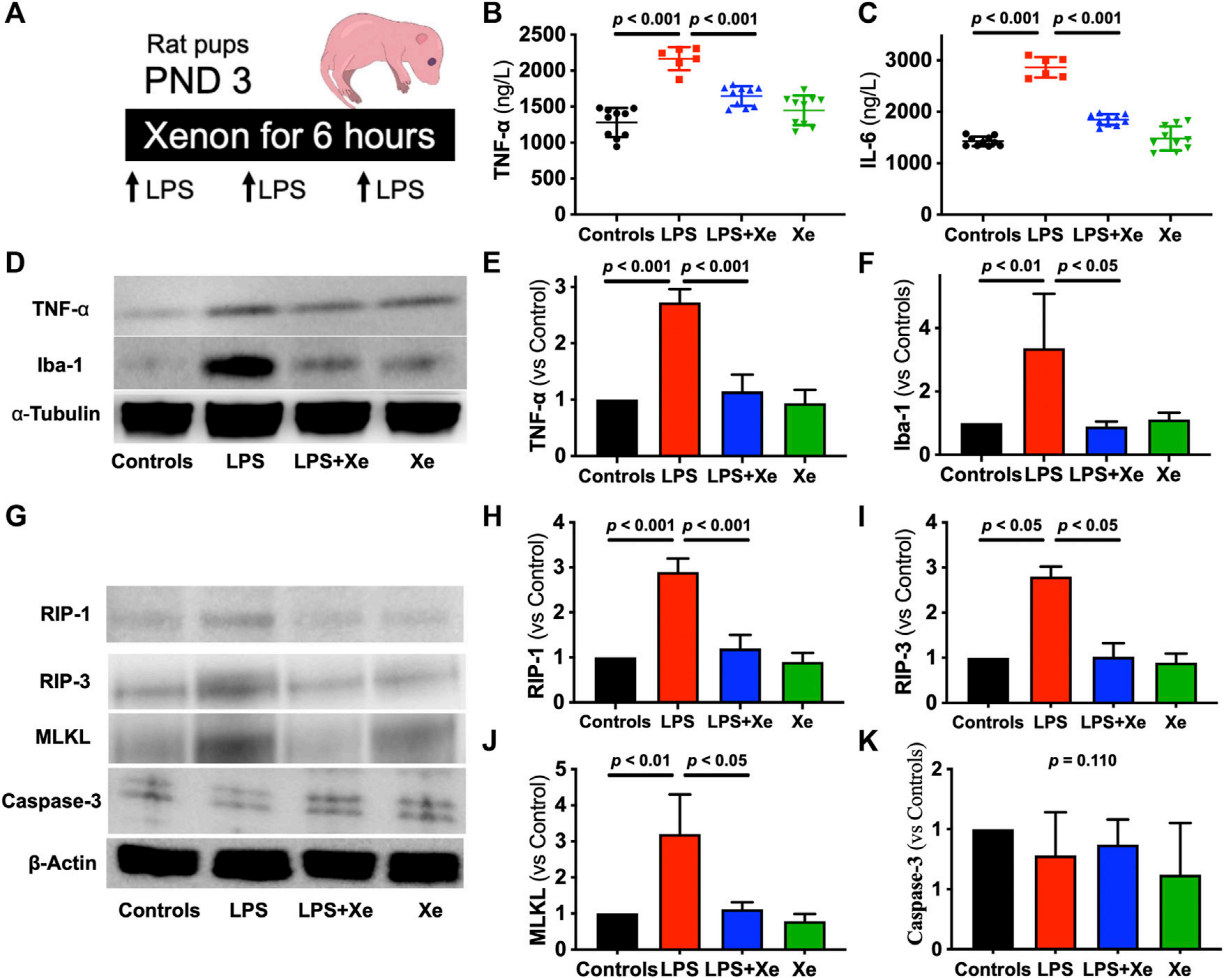
Xenon prevents LPS-caused acute systematic and neuronal inflammation in rat pups. Study Scheme **(A)**. Plasma level of TNF-𝛂 **(B)** and IL-6 **(C)** were measured 6 h after LPS injection plus gas exposure (*n* = 10 in group Con, LPS + Xe and Xe; *n* = 6 in group LPS). Representative western blot images with quantification for acute neuroinflammation **(D–F)** and acute cell death **(G–K)** from the hippocampus. Data are expressed as mean ± SD (*n* = 4 in each group). (Con = saline injection +70%N_2_/30%O_2_; LPS = LPS injection +70%N_2_/30%O_2_; LPS + Xe = LPS inhalation +70%Xenon/30%O_2_; Xe = saline injection +70% Xe/30% O_2_; PND = Post Neonatal Day).

Pregnant Sprague-Dawley rats on gestational day 16 were purchased from Chengdu Dossy Experimental Animals CO., LTD. Each rat was housed in one separated cage and monitored for the offspring’s birth. Except for experiment, all the pups were kept with their mothers till post-natal day 21 (PND 21). Three-day-old Sprague–Dawley rat pups (PND 3) were used in this study. Pups received intraperitoneal (*i.p.*) injection of lipopolysaccharide (LPS, *E. coli* 055:B5, from Sigma, St. Louis, MO, United States) for three times with a 2-h interval between each injection. For the first injection, LPS was given at the dose of 500 mcg/kg while 250 mcg/kg for the rest two injections. This LPS regimen was determined from our pilot study (data not shown). LPS was dissolved in 0.9% normal saline and was given at the volume of 0.1 ml/10 g body weight. The control pups received the same volume of normal saline following the identical protocol (Figure 1A).

Immediately after first injection, the pups were exposed to either 70% xenon or 70% N_2_ balanced with 30% O_2_ for 6 h in a gas-tight chamber coupled with a customized closed-circuit system, in which CO_2_ was absorbed by soda lime. At the second and third LPS injection time points, the pups were quickly removed from the chamber, receiving LPS and were returned into the chamber within 30 s. Pups were kept normothermia by using a heating pad placed inside the chamber. O_2_ and CO_2_ concentrations in the chamber were regularly monitored with an anesthetic gas monitor (Datex-Ohmeda, GE Healthcare, Shanghai, China).

Rat pups of both sexes from each litter were randomly divided to receive one of the following four treatments: Control (Con, *i.p.* saline + N_2_/O_2_); LPS (*i.p.* LPS + N_2_/O_2_); LPS + Xenon (*i.p.* LPS + Xenon/O_2_); Xe alone (Xe, *i.p.* saline + Xe/O_2_).

Immediately after the 6-h gas exposure, 6 pups in LPS group and 10 pups in each other three groups were sacrificed with *i.p.* injection of overdose sodium pentobarbital and blood was sampled via cardiac puncture. The plasm was separated by centrifuging the blood sample at 3,000 rpm for 7 min and kept at −80°C till further analysis. Then, their brains were quickly removed and kept at −80°C till for western blotting use.

The rest of the pups were returned to their dams. On PND 21, all the remaining rats were weaned from their mothers and were housed into different cages for subsequent cognitive assessment (see below).

### Administration of necrostatin-1

Twenty minutes after final *i.p.* LPS injection, rat pups received intracerebroventricular (*i.c.v.*) injection of 4 μg necrostatin-1 (Nec-1 from from Sigma, St. Louis, MO, United States) dissolved in 4 μl saline with 5% DSMO. The intracerebroventricular injection was performed as previously described (Ma et al., 2016). In brief, pups were placed in a stereotactic frame with a neonatal rat adapter. Under light isoflurane anesthesia, a 5-μl Hamilton micro syringe needle (Hamilton Company, United States) was inserted at the location of 2.0 mm posterior and 1.5 mm left to the bregma, and 2.5 mm deep to the skull surface. The syringe was kept in place for 10 min after completion of injection to prevent liquid reflux. 4 μl saline with 5% DSMO was injected as control (Figure 4A).

Rat pups were randomly divided to receive the one of the following three treatments: Control (*i.p.* saline + *i.c.v.* saline with 5% DSMO); LPS (*i.p.* LPS + *i.c.v.* saline with 5% DSMO); LPS + Nec-1 (*i.p.* LPS + *i.c.v.* Nec-1).

After last LPS injection, all the rest pups were returned to their dams and were weaned from their mothers on PND 21 for further cognitive assessment.

### Cognitive assessment

**FIGURE 2:**
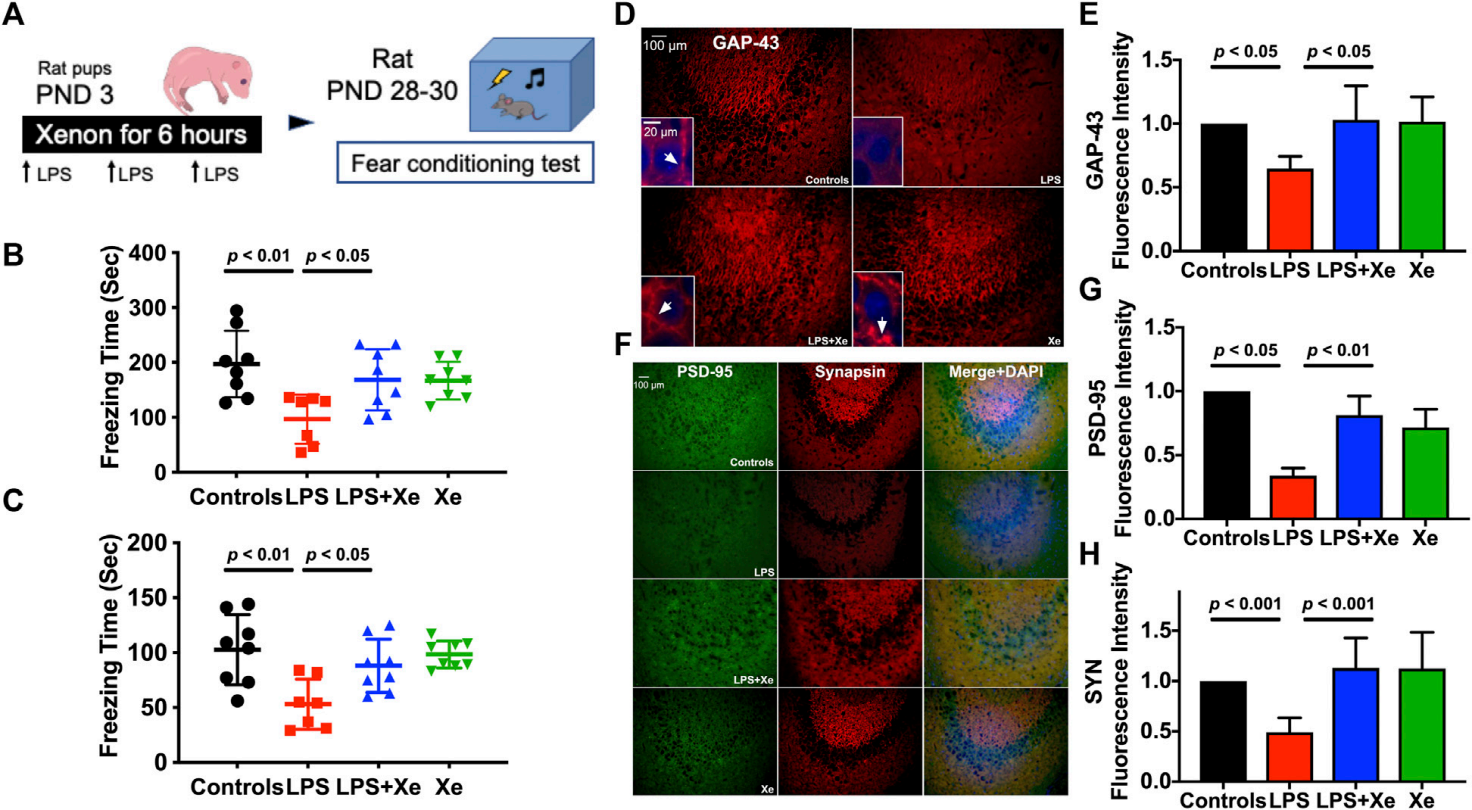
Xenon attenuated juvenile cognitive deficit and preserved synaptic integrity after LPS administration at neonatal stage. Study Scheme **(A)**. Freezing time for context test **(B)** and cue-tone test **(C)** tested 26 days and 27 days after initial LPS injection, respectively. Data are expressed as mean ± SD. (*n* = 7 in group LPS and *n* = 8 in group Con, LPS + Xe and Xe). Representative immunohistochemistry staining **(D)** with quantification **(E)** for hippocampal expression of Growth Associated Protein 43 (GAP-43) in PND 30 rats (27 days after initial LPS injection). The arrow heads in the insets showed the accumulation of GAP-43, which is essential for functional axon growth. Representative immunohistochemistry staining **(F)** with quantification **(G,H)** for hippocampal expression of Synapsin and PSD-95 in PND 30 rats (27 days after initial LPS injection), the markers for functional presynaptic terminals and excitatory postsynaptic membranes respectively. Data are expressed as mean ± SD and *n* = 4 in each group. (Con = saline injection + 70%N_2_/30%O_2_; LPS = LPS injection + 70%N_2_/30%O_2_; LPS + Xe = LPS inhalation + 70%Xenon/30%O_2_; Xe = saline injection + 70% Xe/30% O_2_).

On PND 28 (25 days after LPS treatment), Figure 2A all the remaining rats were subjected to fear conditioning assay, which is a reliable assessment for both hippocampal-dependent and independent memory, as previously reported (Pugh et al., 1998). Rats were trained and tested individually in the fear conditioning chamber (ANY-maze, IL), with an electrified grid floor. Background noise of 65 dB was presented as long as the rat was inside the chamber, unless otherwise notified. The activity of the rat inside the chamber was recorded continuously by a camera mounted to the ceiling of the chamber. Rats were transferred to the testing room 30 min before training/testing began for habituation. Between each training/testing session, the chamber was thoroughly cleaned with a towel soaked in 70% ethanol.

On PND 28 (training session), rats received 15 pairs of conditional and unconditional stimuli at an interval of 57.5 s. The 2.5 kHz cue tone at 75-dB (conditional stimulus, CS) was played for 2.5 s, which was co-terminated with the unconditioned stimulus (US), a 0.5-mA foot-shock presented at the last 2 s of the cue tone. The rat was removed from the chamber 300 s after the last shock and returned to the home cage.

On PND 29 (context test session), the trained rat was re-placed into the identical chamber for 10 min, with the same acquisition environment but no CS or US. The freezing behavior is defined as complete absence of any physical movement except respiratory movement. The freezing time during the 10-min study period was determined via off-line recorded video analysis by a researcher who was unaware of the experimental protocol.

On PND 30 (cue-tone test session), the rat was placed in a novel chamber with completely different context of the side walls, lighting color, shape of the ceiling, and the olfactory cues. After 5-min inhabitation, five cycles of CS (the identical 2.5 kHz sound at 75 dB) were given for 2.5 s followed by an interval of 57.5 s. The freezing time during the 5-min observational period was determined as described above.

Upon finishing the cognitive assessments, the rats were sacrificed with i.p. injection of overdose sodium pentobarbital. Half of these rats were perfused with 4% paraformaldehyde (PFA) in phosphate-buffered saline (PBS). Brains were then removed and fixed in the 4% PFA for at least 24 h before being further processed for immunohistochemistry staining. For the rest half, the brain was quickly removed, and the hippocampus was dissected, which was then snap-frozen in liquid nitrogen and stored at −80°C till further processing for western blotting.

### Immunohistochemistry staining

The post PFA fixated brain samples were dehydrated in 30% sucrose. Thereafter, 20-μm coronal sections were made with a cryomicrotome. Sections were incubated with a blocking solution of 5% donkey serum and 0.3% Triton in PBS for 30 min at room temperature and then incubated with the following primary antibodies overnight at room temperature: rabbit anti-GAP 43 (1:200; Abcam), mouse anti-NeuN (1:200; Millipore), rabbit anti-Synapsin (1:200; Cell Signaling), goat anti-PSD 95 (1:200; Abcam), rabbit anti-RIP1 (1:200; Abcam), and rabbit anti-RIP 3 (1:200; Abcam), respectively. On the next day, sections were incubated with Alexa Fluor^®^ 488 or 594 conjugate secondary antibodies (Invertrogen) for 1 h at room temperature. Finally, the sections were mounted with ProLong^®^ Gold Antifade Reagent with DAPI (Invertrogen). Images were acquired with a Zeiss immunofluorescence microscope under constant exposure time for the same set of markers among the groups. Immunofluorescence was quantified using ImageJ (U.S. National Institutes of Health, Bethesda, MD, United States). Ten representative regions per section were randomly selected by an assessor blinded to the treatment groups. Fluorescence intensity was normalized against the controls and was expressed as the ratio relative to the control animal.

### Western blotting

The hippocampus samples were homogenized in cell lysis buffer (Cell Signaling, Danvers, MA, United States), with a sonicator. The samples were then centrifuged at 12, 800 r.p.m. for 30 min at 4°C, and the supernatant was collected. Total protein concentration was quantified using the Bradford protein assay (Beyotime Biotechnology, Shanghai, China). Equal amount of protein extracts was heated, denatured, and separated by electrophoresis on a NuPAGE 4%–12% Bis-Tris gel (Invertrogen, Waltham, MA). Due to the impaired supply chain during the COVID-19 pandemic, some of the electrophoresis was performed with handcasting SDS-PAGE gels. The proteins were then transferred to polyvinylidene difluoride membranes, which were blocked for 90 min at room temperature, in 5% non-fat powdered milk in 0.1% Tween 20/tris-buffered saline (TBST) and were probed with the following primary antibodies: rabbit anti-TNF-α (1:1000; Abcam), rabbit anti-Iba1 (1:1000; Wako), rabbit anti-RIP-1 (1:750; Abcam), rabbit anti-RIP-3 (1:750; Abcam), rabbit anti-MLKL (1:750; Abcam), rabbit anti-β-actin (1:1,000; Cell Signaling), mouse anti-α-tubulin (1:2,000; Sigma) and cleaved caspase-3 (1:500, Cell Signaling), respectively, in 5% non-fat dry milk in TBST overnight at 4°C, followed by HRP-conjugated secondary antibodies (Cell Signaling) for 1 h. Finally, the membranes were visualized with an enhanced chemiluminescence detection kit (Santa Cruz, TX, United States). The protein bands were captured with an image processor (Amersham Imager 600, GE Healthcare Life Sciences, Shanghai, China) and their intensities were measured by using ImageJ (National Institutes of Health, Bethesda, MD, United States). Results were normalized to levels of the housekeeping protein β-actin or α-tubulin and were expressed as ratio relative to the control for data analysis.

### Enzyme-linked immunosorbent assay

Plasm levels of TNF-α and IL-6 were determined by ELISA. The assay kits were purchased from Neobioscience (Neobioscience Biotechenologies, Shanghai, China) and the analysis was performed following the manufacture’s instruction.

### Statistical analysis

All numerical data were expressed as mean ± standard deviation (SD). Comparison between the study groups was analyzed with one-way analysis of variance (ANOVA), followed by *post hoc* Student Newman-Keuls test as necessary. A *p* < 0.05 was considered statistically significant. All analysis was performed with GraphPad Prism (GraphPad Software, La Jolla, CA, United States).

### Results

#### Xenon attenuated LPS-induced acute systemic inflammation and neuronal necroptosis, but not apoptosis

Repeated LPS injection caused transient sickness behaviors in neonatal rat, such as lack of normal movement for breastfeeding. Four out of 11 pups in the LPS group died in the first 24 h after LPS administration while there was no animal death in the other three groups (*p* = 0.0226, Supplementary Figure S1). Measured at the end of the 6-h gas exposure (2 h after the third LPS injection), plasma levels of TNF-α and IL-6 increased significantly in the survived pups received LPS + N2/O2 (*p* < 0.001 for both TNF-α [1278 ± 202 vs. 2166 ± 161 ng/L] and IL-6 [1427 ± 92 vs. 2866 ± 198 ng/L]). Xenon inhalation effectively prevented the burst of plasma TNF-α (1646 ± 138 ng/L) and IL-6 (1849 ± 103 ng/L) levels, as showed in Figures 1B,C.

In line with the change in systemic inflammation, LPS resulted in activation of neuroinflammation, as the hippocampal expression of TNF-α and Iba-1 (the marker of microglia activation under neuroinflammation) was significantly elevated in pups received LPS + N_2_/O_2_ (*p* < 0.001 for TNF-α; *p* < 0.01 for Iba-1, Figures 1D–F). Xenon inhalation also inhibited the expression of both TNF-α and Iba-1 in hippocampus.

Next, we investigated the cell death in the hippocampus (Figure 1G). LPS caused over-expression of RIP-1 (Figure 1H, *p* < 0.001), RIP-3 (Figure 1I, *p* < 0.05) and MLKL (Figure 1J, *p* < 0.01), the markers for necroptosis, the type of cell death that is closely related with inflammation. Interestingly, another type of cell death, apoptosis, was not activated in neonates receiving LPS (Figure 1K, *p* = 0.110).

#### Xenon attenuated juvenile cognitive deficit and improved synaptic integrity after neonatal LPS administration

Fear-conditioning test was initiated 25 days after LPS injection. As showed in Figures 2B,C, LPS injections at PND 3 resulted in a significant decrease in freezing-time, both in context (*p* < 0.01) and cue-tone test sessions (*p* < 0.01). Xenon at 70% significantly prevented LPS exposure-induced cognitive dysfunction in juvenile rats (*p* < 0.05 for LPS vs. LPS + Xe in both context and cue-tone test).

As early post-natal life is of vital importance for synaptogenesis, we further investigated several key components of synapse in the hippocampal CA3 region: axonal growth associated protein 43 (GAP-43), synapsin (SYN) for presynaptic terminals and PSD-95 for excitatory postsynaptic membranes. In line with behavioral data, neonatal LPS administration led to a decreased hippocampal GAP-43 expression when assessed 25 days after initial LPS injection. Xenon treatment restored GAP-43 expression in juvenile rats (*p* < 0.05) as showed in Figures 2D,E. The synaptic integrity was further assessed by examining the expression of SYN and PSD95. LPS exposure resulted in a decreased expression of both markers, which were also restored by xenon inhalation (*p* < 0.01 for PSD-95; *p* < 0.001 for SYN, Figures 2F–H).

#### Xenon prevents persistent activation of necroptosis in juvenile rats with neonatal LPS administration

**FIGURE 3:**
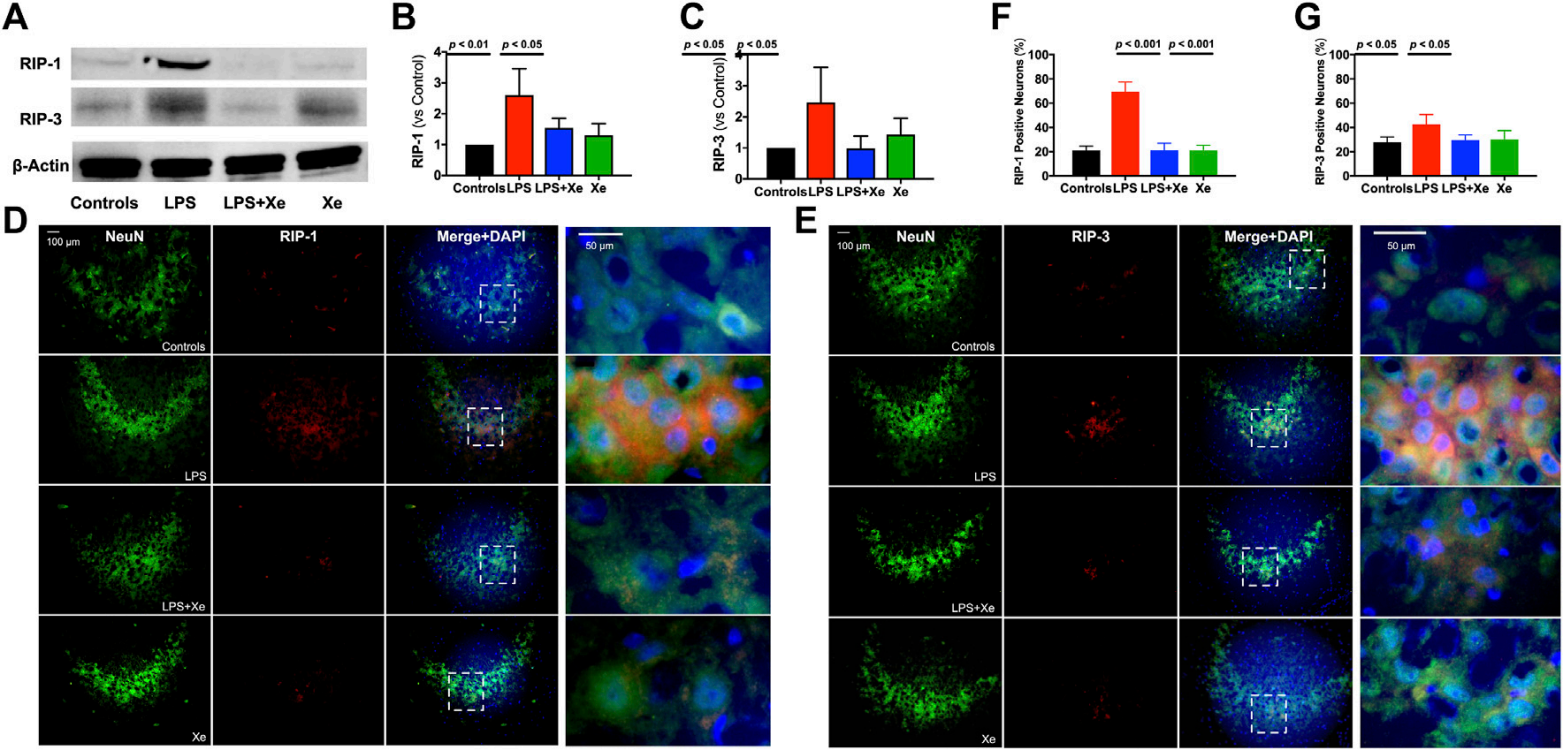
Xenon prevented persistent activation of necroptosis in juvenile rats with neonatal LPS administration. Representative western blot bands **(A)** with quantification **(B, C)** for necroptosis in PND 30 animals (27 days after initial LPS injection). Representative immunohistochemistry staining **(D, E)** with quantification **(F, G)** indicting persistent neuronal necroptosis in PND 30 animals. Data are expressed as mean ± SD. (Con = saline injection +70%N_2_/30%O_2_; LPS = LPS injection +70%N_2_/30%O_2_; LPS + Xe = LPS inhalation +70%Xenon/30%O_2_; Xe = saline injection +70% Xe/30% O_2_; n = 4 in each group except n = 3 in group LPS for western blotting).

27 days after initial LPS challenge, western blotting analysis indicated that necroptosis remained active in the hippocampus, as showed in Figure 3A. The expression of both RIP-1 and RIP-3 were significantly increased in juvenile rats’ hippocampus with neonatal LPS exposure (*p* < 0.01 for RIP-1, Figure 3B; *p* < 0.05 for RIP-3, Figure 3C). Xenon inhalation significantly inhibited the expression of both RIP-1 and RIP-3. Next, the evidence of necroptosis in hippocampal CA3 neurons was investigated. As showed in Figures 3D,E, more than 60% of neurons were positive for RIP-1 staining while more than 40% were positive for RIP-3 staining in juvenile rats’ hippocampus with neonatal LPS injection, both of which were significantly higher than those in the control animals (*p* < 0.001 for RIP-1-positive CA3 neurons, Figure 3F; *p* < 0.05 for RIP-3-positve CA3 neurons, Figure 3G). In line with the western blotting data, xenon inhalation significantly decreased both RIP-1- and RIP-3-positive hippocampus CA3 neurons.

#### Effect of necroptosis inhibition on juvenile cognitive function following neonatal LPS administration

**FIGURE 4:**
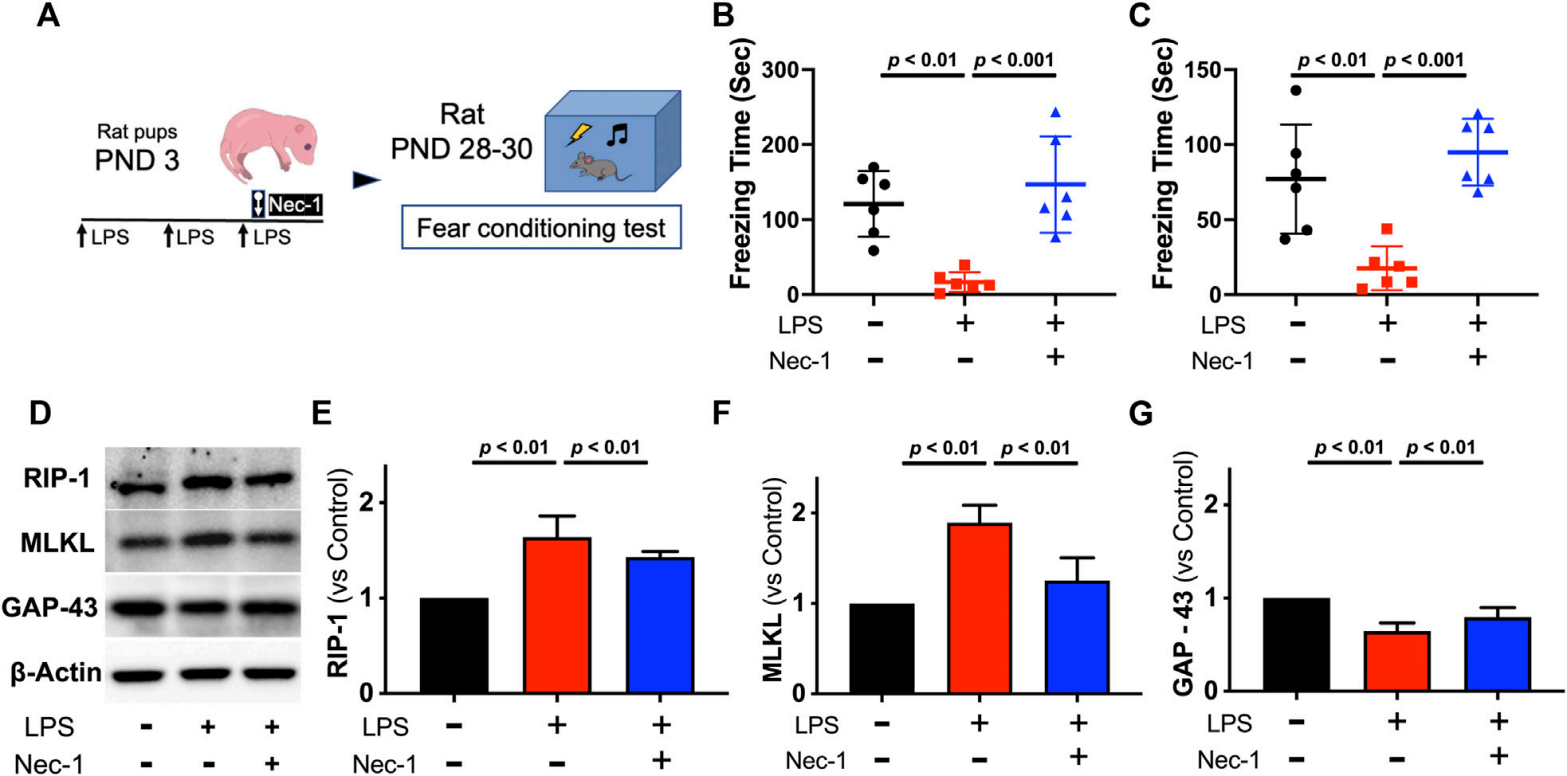
Necrostatin-1 also improved cognitive function and prevented necroptosis activation in juvenile rats with neonatal LPS administration. Study Scheme **(A)**. Freezing time for context test **(B)** and cue-tone test **(C)** tested 26 days and 27 days after initial LPS injection, respectively. Representative western blot images **(D)** with quantification **(E–G)** for necroptosis from the hippocampus. Data are expressed as mean ± SD Data are expressed as mean ± SD. (Nec-1 = necrostatin-1; *n* = 6 in each group).

To determine whether the neuroprotective effect of xenon resulted from inhibition of necroptosis, RIP-1 inhibitor, necrostatin-1 (Nec-1), was administrated via intracerebroventricular injection to block activation of necroptosis within the brain. Rat pups received Nec-1 had significantly improved cognitive function when tested in juvenile age, as the durations of freezing-time were longer both in context test (*p* < 0.01, Figure 4B) and cue-tone test (*p* = 0.01, Figure 4C). As expected, Nec-1 blocked the expression of RIP-1 (*p* < 0.01, Figures 4D,E) and the downstream MLKL (*p* < 0.01, Figure 4F) caused by neonatal LPS exposure while restored expression of GAP-43 (*p* < 0.01, Figure 4G).

### Discussion

In this study, we demonstrated that repeated LPS administration in rat pups resulted in cognitive deficit in juvenile rats and impaired synapse integrity, reflected as significantly decreased expression of key synaptic structure proteins. It was further demonstrated that this neurodevelopment deficit can be attenuated with xenon inhalation via modulation of necroptotic cellular signaling pathway.

Neonatal sepsis induced detrimental effects on vulnerable immature brain, which finally leads to late-onset neurodevelopment impairments, such as deficits in neuropsychologic performance and cognitive difficulties in juvenile or early adult life (Als et al., 2013). It is generally accepted that acute systemic inflammatory response plays a vital role in sepsis-induced solid organ injuries, including the brain (Cardoso et al., 2015). However, it remains unclear how this acute neuroinflammation induces long-term neurological functional changes in central nervous system (CNS). Recent study showed that early postnatal LPS exposure led to impaired communicative functions in rats, and decreased neuronal apoptosis and hence impaired neuroplasticity for advanced functional development (Pang et al., 2016). Our results also failed to find any increased apoptosis in rat hippocampus after neonatal LPS injection. Considering the importance of naturally occurring apoptosis in normal neurodevelopment (Nijhawan et al., 2000), it is therefore rational for us to hypothesize that other types of neuron death secondary to initial neuroinflammation may account for neonatal sepsis-induced neurofunctional impairment.

In line with previous studies, LPS injection resulted in a burst release of TNF-α both in blood and hippocampus. In addition to causing acute inflammation, TNF-α has been identified as the inducer for necroptosis (He et al., 2009). As programmed cell death of its kind in addition to apoptosis, necroptosis shares common morphological features with necrosis while it is tightly regulated by kinases, such as RIP-1 and RIP-3 (Zhang et al., 2009). There is a growing body of evidence showing that necroptosis may be a common feature of neuronal death in multiple CNS diseases, such as neurodegenerative diseases (Caccamo et al., 2017) and traumatic brain injury (You et al., 2008). Recent studies further indicated that activation of RIP-1/RIP-3 kinase was involved in the pathogenesis of neonatal hypoxia-ischemia encephalopathy (Northington et al., 2011).

In this study, RIP-1 and RIP-3 expression in the hippocampus was significantly increased after LPS injection. Furthermore, necroptotic cell death process remained active 27 days after initial LPS insult. It is highly possible that this persistent necroptosis leads to neuronal death and cognitive impairments. With early intracerebral injection of Nec-1 to inhibit the cerebral expression of RIP-1 after LPS administration, cognitive function was significantly improved as we demonstrated in our study. This novel finding may suggest that targeting necroptotic neuronal cell death could be an intervention strategy to tackle sepsis-induced the long-term brain damage. In line with our hypothesis, recent study has revealed that RIP-3, but not RIP-1, plays an indispensable role in myocardial ischemic-reperfusion injury (Zhang et al., 2016). However, further studies are needed to identify whether one or both RIP molecules participate in the necroptotic pathway that is responsible for neonatal sepsis-induced neurodevelopment impairment.

The noble gas xenon has been well documented for its potent protective effect against multiple types of brain injuries, including ischemic (Schmidt et al., 2005), hypoxic (Ma et al., 2006), traumatic (Campos-Pires et al., 2015), and neuroinflammation-induced brain injury, such as post-operative cognitive dysfunction (Ma et al., 2003). Here, we showed that early inhalation of xenon protected against neonatal LPS exposure-induced late-onset cognitive dysfunction in juvenile rats. Previous studies have demonstrated the anti-inflammatory effect of xenon (Abramo et al., 2012); therefore, the protection might result from early suppression of inflammation as we found that xenon did inhibit activation of both systematic and neuro-inflammatory response following LPS injection. Our results furthermore suggested that xenon produced neuroprotection via inhibition of necroptosis, as inhibition of necroptosis either by xenon or selective necroptosis inhibitor (Nec-1) produced the same neuroprotective effect in our neonatal rat sepsis model. Other unknown mechanisms might also contribute. For example, xenon has been demonstrated to protect LPS-induced renal injury via upregulation of microRNA-21 (Jia et al., 2015).

Our study is not without limitations. Firstly, there have been debate on whether LPS-induced inflammatory response could be considered as sepsis, since LPS, as the endotoxin produced by Gram-negative bacteria, produces only transient “toxemia” but not bacteremia (Schwarz and Bilbo, 2011). However, in most sepsis patients, the brain was injured due to the cytokines entering CNS (Flierl et al., 2010), but not direct bacterial infection in the CNS (Strunk et al., 2014). Therefore, neuroinflammation resulted from LPS-induced massive cytokines release is comparable to that observed in septic patients with cerebral complications. In addition, repeated LPS administration could be regarded to be septic state, which produced more severe and persistent inflammatory response than single-dose injection, as suggested by other researchers (Püntener et al., 2012). Thus, we believe that the inflammation induced with repeated LPS injection produces injuries comparable to that in clinical settings (Camacho-Gonzalez et al., 2013). Secondly, the cecum ligation and puncture (CLP) procedure was not used in this study to create a sepsis model. However, one can appreciate that the neonatal rat pup is too small and too fragile to tolerate that procedure. Thirdly, xenon inhalation was initiated immediately after LPS insulation. With regard to recent xenon-related human trials, the timing of xenon inhalation seems to be of vital importance for improving patients’ outcomes (Azzopardi et al., 2016). Further studies should focus on defining the effective therapeutic window for using xenon to prevent neonatal sepsis-induced neurodevelopment impairment. Lastly, only one concentration of 70% xenon was used in this study. Concentration-dependent response needs to determine minimal effective concentration of xenon in future study for guiding clinical trials.

### Conclusion

Xenon attenuated neurodevelopmental impairment caused by early post-natal LPS challenge, *via* modulation of necroptosis pathway and protecting synopsis. Our preclinical data warrant further research, especially clinical trials, since the feasibility and safety of using xenon in newborns have been confirmed in previous human studies. Our study also provides evidence that targeting necroptosis might be an intervention strategy in preventing neonatal sepsis-induced neurodevelopmental impairment.

## Data Availability

The original contributions presented in the study are included in the article/Supplementary Materials, further inquiries can be directed to the corresponding authors.

## Supplementary material

SUPPLEMENTARY FIGURE S1 Repeated LPS injection was administrated at Post-natal Day 3 (PND 3). There were 4 of 11 rat pups (36%) died within the first 24 h after LPS administration, while no animal death in any other groups. The survival rate in rats receiving LPS+Xe was significantly higher than that in animals receiving LPS alone, as indicated by Kaplan-Meier analysis. (Con = saline injection + 70%N2 / 30%O2; LPS = LPS injection + 70%N2 / 30%O2; LPS+Xe = LPS inhalation + 70%Xenon / 30%O2; Xe = saline injection + 70% Xe/30% O2).
