# Corrigendum: Xenon attenuated neonatal lipopolysaccharide exposure induced neuronal necroptosis and subsequently improved cognition in juvenile rats

**DOI:** 10.3389/fphar.2024.1438335

**Published:** 2024-07-10

**Authors:** Zhimin Liao, Xiaofeng Ou, Cheng Zhou, Daqing Ma, Hailin Zhao, Han Huang

**Affiliations:** ^1^ Department of Anesthesiology, Key Laboratory of Birth Defects and Related Diseases of Women and Children, West China Second University Hospital, Sichuan University, Chengdu, China; ^2^ Department of Anesthesiology and Translational Neuroscience Center, West China Hospital, Sichuan University, Chengdu, China; ^3^ Anaesthetics, Pain Medicine, and Intensive Care, Department of Surgery and Cancer, Faculty of Medicine, Imperial College London, Chelsea and Westminster Hospital, London, United Kingdom

**Keywords:** neonatal sepsis, xenon, neurodevelopmental impairment, necroptosis, neuroinflammation

In the published article, there was an error in Figure 3 as published. The immunoblotting band for **β**-actin in Figure 3A was not correct. The corrected Figure 3 and its caption appear below.

**FIGURE 3:**
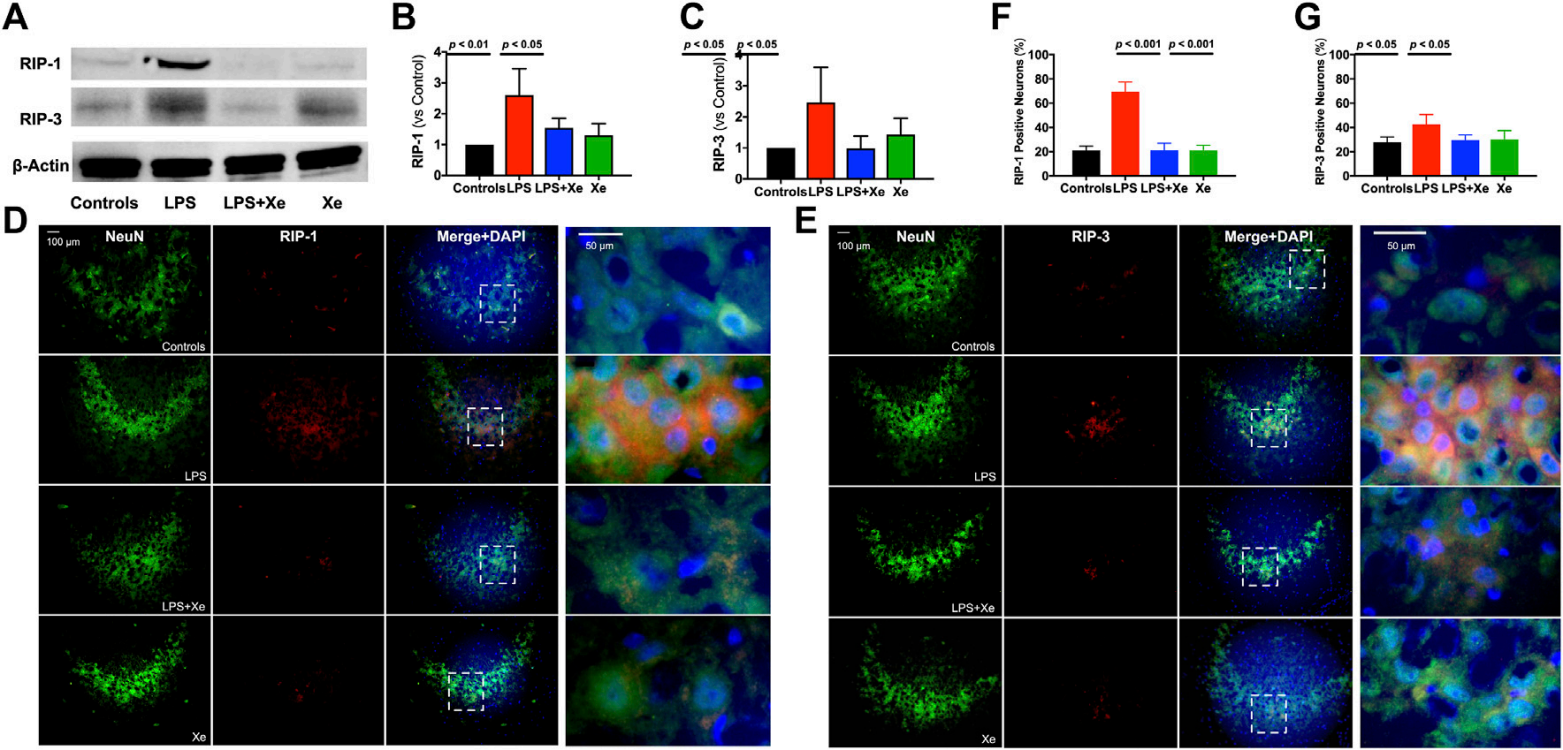
Xenon prevented persistent activation of necroptosis in juvenile rats with neonatal LPS administration. Representative western blot bands **(A)** with quantification **(B, C)** for necroptosis in PND 30 animals (27 days after initial LPS injection). Representative immunohistochemistry staining **(D, E)** with quantification **(F, G)** indicting persistent neuronal necroptosis in PND 30 animals. Data are expressed as mean ± SD. (Con = saline injection +70%N_2_/30%O_2_; LPS = LPS injection +70%N_2_/30%O_2_; LPS + Xe = LPS inhalation +70%Xenon/30%O_2_; Xe = saline injection +70% Xe/30% O_2_; n = 4 in each group except n = 3 in group LPS for western blotting).

The authors apologize for this error and state that this does not change the scientific conclusions of the article in any way. The original article has been updated.

