# Supplementary figures and images for: Xenon attenuated neonatal lipopolysaccharide exposure induced neuronal necroptosis and subsequently improved cognition in juvenile rats
Source: Front Pharmacol. 2022 Dec 2;13:1002920. doi: 10.3389/fphar.2022.1002920 (PMC9755588; doi:10.3389/fphar.2022.1002920)

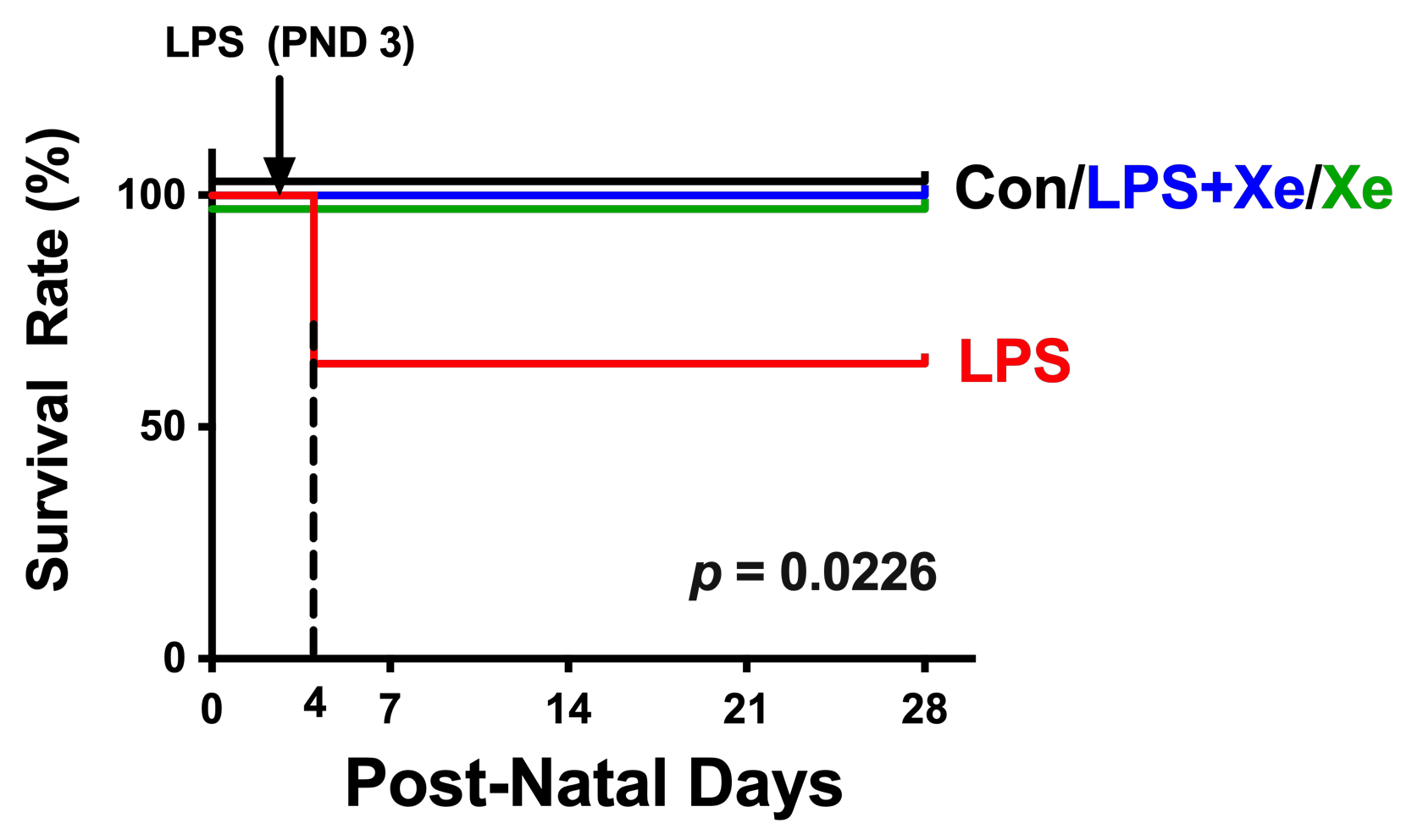

Supplement: Supplementary file 1 [file Image1.TIFF]
